# Clarifying and extending our understanding of problematic pornography use through descriptions of the lived experience

**DOI:** 10.1038/s41598-023-45459-8

**Published:** 2023-10-24

**Authors:** Campbell Ince, Leonardo F. Fontenelle, Adrian Carter, Lucy Albertella, Jeggan Tiego, Samuel R. Chamberlain, Kristian Rotaru

**Affiliations:** 1 School of Psychological Sciences, Monash University, 770 Blackburn Rd, Clayton, VIC 3168 Australia; 2 Turner Institute for Brain and Mental Health, Monash University, Clayton, Australia; 3 Institute of Psychiatry, Federal University of Rio de Janeiro, Rio de Janeiro, Brazil; 4 D’Or Institute for Research and Education (IDOR), São Paulo, Brazil; 5 Monash Biomedical Imaging, Monash University, Clayton, Australia; 6 Department of Psychiatry, Faculty of Medicine, University of Southampton, Southampton, UK; 7 Southern Health NHS Foundation Trust, Southampton, UK; 8 Monash Business School, Monash University, Clayton, Australia

**Keywords:** Psychology, Addiction

## Abstract

Problematic pornography use (PPU) is a complex and growing area of research. However, knowledge of the PPU lived experience is limited. To address this gap, we conducted an online qualitative study with 67 individuals who self-identified as having problematic pornography use (76% male; *M*_age_ = 24.70 years, *SD* = 8.54). Results indicated several dimensions that have not been fully explored in the literature. These included various mental and physical complaints following periods of heavy pornography use, sexual functioning deficits with real partners, and a subjectively altered state of sexual arousal while using pornography. Moreover, we expanded on current knowledge regarding the inner conflict associated with PPU and clarified the ways that users can progress to increasingly intensified patterns of pornography use, such as tolerance/escalation and pornographic binges. Our study highlights the complex and nuanced nature of PPU and provides suggestions for future research and clinical practice.

## Introduction

The use of Internet pornography has become increasingly widespread over the past two decades^1^, providing sexual stimulation for many users^2^. However, up to 10% of users may develop problematic pornography use (PPU)^3^, characterised by impaired control over the behaviour despite negative consequences in important areas of life, including work and relationships^4,5^. Individuals with PPU typically experience difficulties breaking from the pornography-masturbation-orgasm cycle^6,7^, which is experienced as highly reinforcing and eventually tied to maladaptive wanting (clinically experienced as craving)^3,4,6^.

Despite accelerated scientific inquiry into PPU over the past decade, existing research has primarily focused on theoretical understandings of this issue, with relatively few studies directly investigating the lived experiences of people affected by PPU^6–8^. As a result, our current understanding of PPU may not fully capture the factors that characterise, drive, and maintain this problem.

Indeed, a growing body of exploratory literature describes clinically significant features that are not traditionally linked with problematic sexual behaviours. Observations from online PPU recovery communities, peer support groups, and clinical reports document various understudied phenomena, including: (i) cognitive-affective, social, and physical difficulties following heavy periods of pornography use^6,7,9–11^; (ii) sexual dysfunction with real partners as a function of excessive pornography use^7,9,10,12,13^, (iii) and a subjectively altered state of sexual arousal when masturbating to pornography, potentially characterised by diminished pleasure and a trance-like dissociative state^3,4,14,15^.

However, descriptions of these phenomena have primarily emerged in an ad-hoc fashion (i.e., incidentally contained within broader discussions on PPU), rather than through targeted investigations. Although pornography-related sexual dysfunction has received growing scientific attention, results are generally equivocal and the potential links between PPU and sexual functioning problems are not well understood^16–18^. Targeted research into these various under-studied dimensions is therefore timely.

Distress and inner conflict in PPU are key features that also require further attention and research. These psychological processes are complex and multifaceted, but are crucial for effective diagnosis and treatment^4,19^. Although the links between impaired behavioural control and distress are well understood through the lens of compulsive sexual behaviour disorder (CSBD)^4,20^, other forms of inner conflict related to PPU require further exploration.

Moral incongruence theory offers another perspective on inner conflict that has attracted increased attention^19,21^. According to this framework, individuals with traditional religious or sociocultural values that prohibit or discourage pornography use may be particularly prone to inner conflict and distress over their pornography use, even at low levels of engagement^19,22^. However, recent studies have challenged this narrow definition of moral incongruence, suggesting that users can morally object to their pornography use because of other concerns beyond religiosity or conservatism, including concerns over sexual exploitation and negative impacts on relationships^23,24^. Recent work also confirms that moral incongruence can coexist with objectively dysregulated pornography use^25^, complicating efforts to understand the associated underlying psychological mechanisms^21,26^. A deeper understanding of the multifaceted nature of inner conflict and distress will therefore help enhance diagnostic and personalised treatment approaches^3,27^.

How best to measure increasingly intensified pornography use is another area lacking research. Although users can harness the Internet’s unlimited supply of sexual novelty for sexual discovery and experimentation^2^, viewers can also readily offset habituation effects by increasing the duration or frequency of sessions (quantitative escalation), engage with increasingly provocative content (qualitative escalation), fast-moving compilation videos, and/or frequently move between pre-loaded tabs (‘tab-jumping’)^4,7,10,25^. Reports have also emerged regarding ‘edging’ in which the user scrolls through many stimuli while delaying climax, which can become the basis of hours-long pornographic binges^6,28,29^.

These usage patterns each reflect an increasing intensity of pornography use (herein, ‘intensity indicators’) and offer more nuanced information than simply gauging overall quantity of use^30^. Although these intensity indicators remain under-explored^28,31^, such usage patterns have been anecdotally linked to the various under-studied phenomena described above (especially pornography-related sexual dysfunction and acute adverse after-effects)^7^. However, the relationship between these intensity indicators and PPU’s potentially unique features is still largely unknown. Moreover, intensity indicators such as tolerance/escalation and binge patterns may provide insight into the most suitable conceptualisation of PPU, as they are also recognised features of substance and behavioural addictions^32,33^.

The current study aimed to investigate the experiences of individuals who self-identify as having PPU and to explore its associated features. We focused on potentially unique characteristics of PPU, such as perceived adverse after-effects, offline sexual dysfunction, and subjective changes to the sexual experience while using pornography, none of which are captured by existing theoretical models. In light of these knowledge gaps and urgent calls for qualitative research into PPU^6,28,34,35^, we conducted a rich qualitative investigation using an online sample of individuals who self-identified as having PPU that allowed participants to describe their lived experiences in detail^28,36^. The following research questions guided our investigation:

1. How do individuals who self-identify as having PPU describe their experiences (if any) regarding pornography-related sexual dysfunction, acute adverse after-effects, altered state of sexual arousal, inner conflict, and intensity indicators?
2. How do different patterns of intensity indicators (e.g., tab-jumping, pornographic binges, edging) relate to different PPU dimensions (such as acute adverse after-effects, offline sexual dysfunction, or subjectively altered sexual experiences when using pornography)?
3. Are there any other features that individuals who self-identify as having PPU commonly experience, beyond those identified in the literature?

## Methods

### Participants and procedure

To enrich our analysis, we engaged a purposive sample of people who self-identified as having PPU via PPU recovery communities and topic-irrelevant social media pages. This multi-stream recruitment strategy allowed us to engage a more diverse sample, capturing a broader range of PPU severity and to ensure the sample was not restricted to niche Internet forums or communities^8^. Respondents completed an anonymous, cross-sectional online survey, which was designed to maximise participation and reduce shame or embarrassment when discussing sensitive topics. To better understand the initial responses and enrich our understanding, we allowed for follow-up questions on an opt-in basis. The study was approved by the Monash University Human Research Ethics Committee (project #31836) and was performed in according with the Declaration of Helsinki. As determined by ethics approval (i.e., to provide information on country-specific support services), respondents were limited to Australia, Canada, the United Kingdom, and the United States of America. All participants provided informed consent.

Sample size was determined based on the guidelines of ‘informational power’ for qualitative research^38^, which considers design aspects such as sample specificity, breadth of overall aim, and analysis strategy (e.g., across vs. within cases). With these considerations and in consultation with extant qualitative literature, we initially aimed for a sample size of *N* = 60–80, which was iteratively reviewed throughout data collection. Responses were collected using *Qualtrics Experience Management* platform between May and July 2022. In total, *N* = 225 individuals commenced the online survey. Twelve individuals visited the survey link but did not provide informed consent and so did not participate. After removing eligible respondents who were underage (*n* = 1), did not live in the approved countries of residence (*n* = 62), had not consumed pornography within the past year (*n* = 8), had not experienced negative consequences from their pornography use (i.e., did not qualify as having PPU lived experience; *N* = 74), or provided incomplete responses (*n* = 1), *N* = 67 respondents’ data was retained for analysis. No respondents failed the attention checks embedded in the survey (see File S1), and the median completion time was 30 min.

### Qualitative survey design

Following a literature review of current theory and empirical knowledge (including qualitative studies on the PPU lived experience and quantitative research across clinical, sub-clinical, and general population samples), we designed a comprehensive topic guide focussing primarily on our domains of interest (perceived adverse after-effects, impact on sex life, subjective features of the sexual experience with pornography, and intensity indicators) using neutrally framed prompts and probes to minimise confirmation bias. This process was conducted in consultation with relevant co-authors with domain expertise on qualitative research, PPU, and other impulsive-compulsive and behavioural addictions, and the survey was pilot tested with two psychology graduate researchers. To add depth to our analysis, we assessed our domains of interest by triangulating responses across multiple theoretically relevant dimensions (herein referred to as within-case analysis). Pertinent examples are provided below, and the full topic guide can be found in File S1 (Supplementary Information).

Perceived adverse consequences following pornography use were initially assessed with a binary (yes/no) response to “*Have you ever experienced negative unintended effects in the hours after using pornography?*” and those who responded positively were then asked to “*Please describe these negative/unintended experiences. This might include mental, physical, emotional, or social aspects*” and “*What do you think explains these experiences?*” We also asked whether similar issues had arisen from other sexual activities, whether the respondent had experienced distress related to their pornography use, whether they believed that using pornography is morally or ethically wrong (and why/why not), level of religiosity, and how the participant typically feels after a pornographic binge (if applicable).

We investigated perceived impacts on sex life by asking “*Would you say that your pornography use has impacted your sexual functioning, either positively or negatively? If so, please describe*.” To gauge sexual functioning more holistically, we also asked about sexual functioning across different contexts (i.e., for pornography-assisted masturbation, pornography-free masturbation, and partnered sex) and indicators of generalised hypersexuality (e.g., level of sex drive for online vs. offline behaviours, and the degree to which partnered sex would be an attractive substitute when faced with an urge for pornography).

To explore the subjective consummatory experience associated with pornography use, participants responded to the statement “*When I use pornography, I experience different sensations that I don't get from other sexual activities (e.g., compared to porn-free masturbation or partnered sex)*” on a five-point Likert scale (1 = Strongly disagree, 5 = Strongly agree). Those who responded 4 or 5 then answered an open-ended question, “*In what ways does pornography feel different compared to other sexual behaviours?*” These responses were triangulated with qualitative items regarding sexual functioning with pornography (described above) and a qualitative item related to tab-jumping tendencies (“*How important is sexual novelty when you masturbate to pornography? For example, are you sufficiently aroused by a few images/videos, or do you move frequently between tabs?”*).

We assessed intensity indicators through the average quantity of pornography use (hours per week and duration per sitting), binges (two open-ended questions), the role of kinks/fetishes toward one’s pornography use (one open-ended item), and tab-jumping tendencies (one open-ended item).

### Analytic plan

Qualitative data were analysed with a codebook approach to thematic analysis^39,40^ which was chosen over more popular forms (e.g., reflexive thematic analysis) because of its paradigmatic flexibility and accommodation of both top-down (deductive) and bottom-up (inductive) inference^39,41^. Accordingly, our initial coding template contained four a priori domains of interest (perceived adverse after-effects from using pornography, perceived impact on sex life, descriptions of the consummatory experience with pornography, and intensity indicators) contained within a Microsoft Excel spreadsheet. The coding template was inductively modified as we generated new themes, subthemes (second and third-order themes) and integrative themes (i.e., those spanning multiple themes^39,41,42^). Codes were also permitted to exist in multiple themes (i.e., parallel coding)^41^. We employed a critical realist approach in that participants’ responses were taken to reflect their own lived experience while also acknowledging the social and cultural influences that inevitably shape individual interpretations^43–45^. Given our epistemological positioning and recent developments in the thematic analysis framework, coding reliability estimates were not considered appropriate^41,42,44^.

We conducted extensive within- and across case analyses to enrich our interpretations. Within-case analysis occurred first by reviewing responses across the numerous items of interest (described above). This served to enhance data familiarisation and to consider potentially important contextual factors (e.g., pornographic binges).

Through this process, initial codes were generated following data familiarisation, and consistent patterns were then converted into themes via across-case analysis. Moving between across- and within-case analyses occurred iteratively throughout the coding process. Initial codes and themes were generated by the first author and the coding process was cross-checked by two co-authors experienced in qualitative research (KR and AC).

## Results

**Figure 1:**
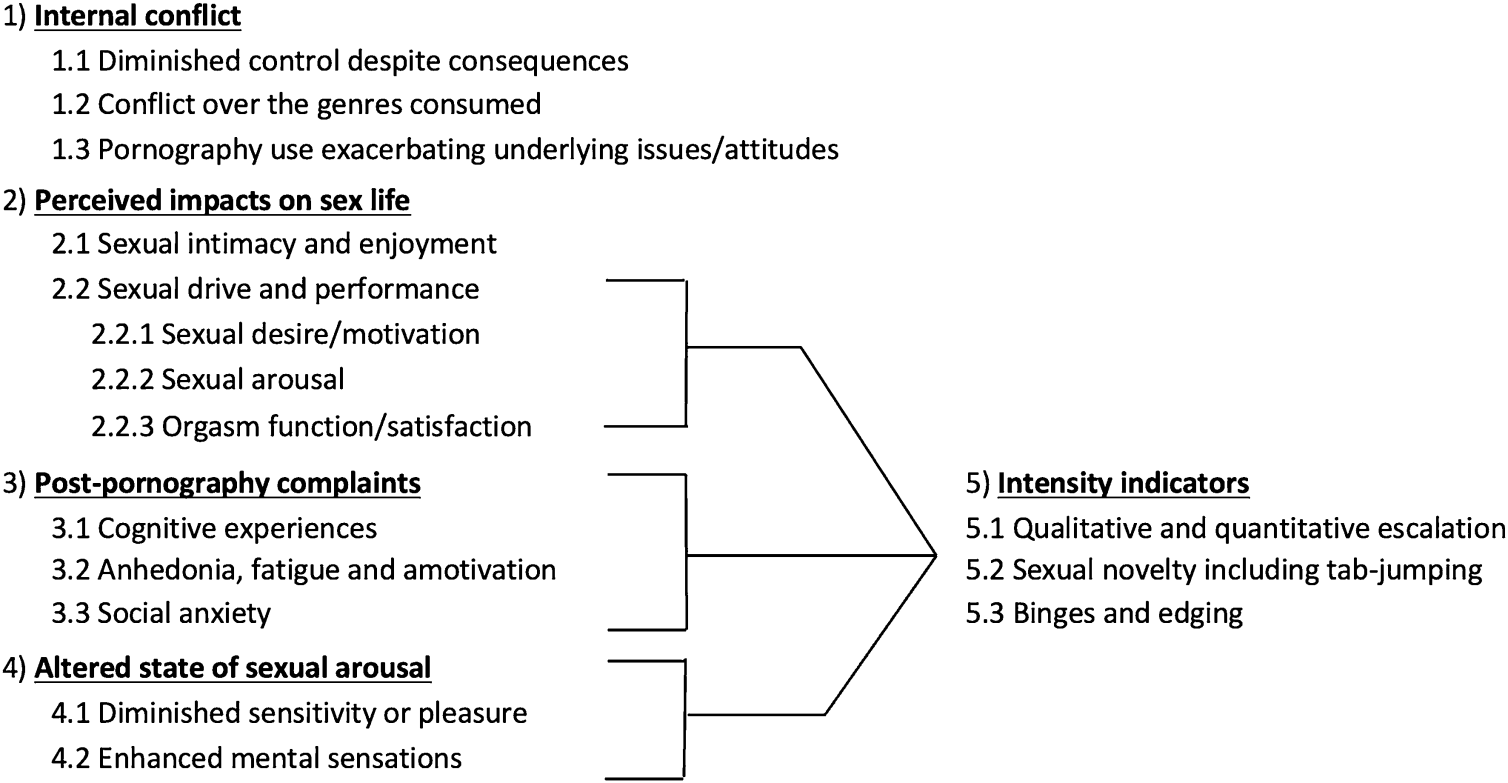
Final thematic structure.

The final sample mainly comprised men aged in their 20 s and 30 s. Sociodemographic information for the final sample is presented in Table 1. Qualitative analysis generated five primary themes: four standalone themes (internal conflict, impact on sex life, perceived adverse after-effects, and uniqueness of consummatory experience) and one integrative theme (intensity indicators). This thematic structure not only subsumed our a priori domains of interest but also captured other dimensions considered informative to the PPU lived experience. The final thematic structure is presented in Fig. 1. Table S1 provides a list of illustrative quotes beyond those presented in-text. Age-sex descriptors (e.g., ‘21 M’ for 21-year-old male) are provided alongside each quote.

**Table 1:** Sociodemographic information.

Variable	Mean (SD)/*n* (%)
Age (years)	24.70 (8.54)
Country of residence	
Australia	31 (46)
Canada	3 (5)
United Kingdom	8 (12)
United States	25 (37)
Sex assigned at birth	
Male	51 (76)
Female	16 (24)
Gender	
Male	50 (75)
Female	15 (22)
Non-binary/gender diverse	2 (3)
Sexual orientation	
Heterosexual	52 (78)
Homosexual	5 (7)
Bisexual	8 (12)
Other	2 (3)
Employment status	
Full time	21 (31)
Part time/casual	6 (9)
Student and not employed	12 (18)
Student and employed	22 (33)
Not employed and not a student	6 (9)
Education	
Primary/elementary/middle school	1 (1)
Secondary/high school	26 (39)
Tertiary	34 (51)
Higher degree	6 (9)
Relationship status	
Single	42 (63)
In a relationship	20 (30)
Married	4 (6)
Divorced/separated	0 (0)
Widowed	1 (1)
Religiosity	2.37 (1.24)

Religiosity rated on a 5-point Likert scale (0 = *Not at all religious*, 4 = *Definitely religious*).

### Theme 1: internal conflict

Many respondents indicated an inner resistance, tension, or distress over their pornography use, collectively referred to here as inner conflict^31^. This took various forms, including conflict from impaired behavioural control despite negative consequences, discomfort over the specific genres of pornography consumed, and paradoxically heightened tension toward one’s pornography use when the behaviour was intended to relieve psychological stress.

#### Subtheme 1.1: conflict from diminished control despite consequences

Participants commonly reported inner conflict, annoyance, and guilt stemming from diminished control over pornography use despite recognised consequences (hereafter, ‘PPU-related distress’). Related consequences were wide-ranging, including negative impacts on mental health, interpersonal relationships, reduced productivity at work or school, religious conflict, and sexual functioning concerns with intimate partners. Additionally, respondents often framed the resulting tension, guilt or shame as addiction-related conflict, reflected in such sentiments as “*Porn feels addictive and I think I carry shame around with it… you feel a sense of guilt and annoyance that your desires control you*” (21 M) and feeling “[I feel] s*hame and disappointment*… *from multiple attempts to quit using porn*” (23 M).

#### Subtheme 1.2: conflict over the genres consumed

Some respondents experienced substantial discomfort regarding the sexual genres previously viewed, which was often described as sexually demeaning, disturbing, or unethical. As one man stated, such content "*crosses many thick ethical red lines*" (29 M). Progressing to these newfound genres did not seem to reflect sexual curiosity, rather processes of escalation whereby “*my tastes have changed and my time spent indulging has increased. I have looked at things that I would have once found disgusting”* (25 M)*,* and *“I used to masturbate to nonextreme stuff* [sic] *but it escalated as I got older”* (22 M).

#### Subtheme 1.3: pornography exacerbating underlying issues/attitudes

Respondents indicated that their pornography use exacerbated other mental and emotional issues, especially when they used pornography to ameliorate low mood or loneliness (i.e., negative reinforcement). Paradoxically, this emotional regulation strategy often intensified the underlying problems, whereby using pornography instead *“adds to my anxiety and depression”* (26 M) or promoted a “*feeling of unsatisfaction* [sic] *because I wasn't with someone*” (20 M).

Others reported an inner conflict from underlying moral, ethical, or religious attitudes that preclude pornography use, indicative of moral incongruence. For instance, one woman described “*feeling restless, guilty, disgusted with myself, bored, lonely,”* which was attributed to "*subconscious attitudes towards porn because I was brought up to think sex was disgusting*" (26F). Such respondents often reported distress from other sexual activities, suggesting a role for broadly restrictive attitudes toward sexual activity. Nearly all such sentiments related to moral/ethical incongruence were reported by female participants, indicating a potential gender effect. Many respondents reporting moral incongruence also endorsed PPU-related distress, indicating that these sources of inner conflict were not mutually exclusive.

### Theme 2: perceived impacts on sex life

Respondents generally indicated that their pornography use had a negative impact on their sexual health and wellbeing. This included reduced sense of connection with sexual partners, as well as reduced libido, ease of arousal, and orgasm functioning.

#### Subtheme 2.1: sexual intimacy and enjoyment

Respondents described links between their pornography use and subsequent decreased quality of sexual intimacy with real partners. For instance, when asked about pornography’s impact on sexual functioning, respondents described feeling “*Less connected when sexually intimate with* [a] *real partner*" (23F) and “*Less in touch with* [my] *partner*” (56 M). Similarly, others described their pornography use shifted their expectations placed upon their partner, whereby “*It* [using pornography] *has created unrealistic expectations of intimacy with my partner*” (22 M) and developing a “*misguided vision of healthy sexual activity*" (24 M).

#### Subtheme 2.2 sexual drive and performance

##### Subtheme 2.2.1: sexual desire/motivation

Respondents also cited reduced sexual drive when offline, often attributed to having achieved sexual gratification through Internet pornography. As one respondent stated, *“I think it makes me comfortable not putting myself out there to find a partner if I know I can pleasure myself sexually with porn"* (20, non-binary). Those already in a romantic relationship also reported reduced sexual desire for their intimate partner because of a preference for Internet pornography, whereby* “*[using pornography] *lowered my sex drive in the past to where I would have rather masturbated to porn sometimes or I wouldn't initiate sexual encounters with my partner”* (34 M). On the other hand, abstaining from pornography seemingly helped to reverse such effects whereby “*If I abstain for a long time from pornography I feel more motivation/need to pursue a partner"* (25 M)*.* There were also no indications of sexual compulsivity toward real partners, and only a minority indicated an interest in offline masturbation or partnered sex when faced with an urge for pornography, inferring a sexual reliance toward Internet pornography rather than generalised hypersexuality.

##### Subtheme 2.2.2: sexual arousal

When directly asked about pornography’s impact on sexual functioning (either positive or negative), respondents often indicated strong negative outcomes. This was reflected by such remarks as “*negatively* [impacted]*, reduced sensitivity and more difficult to get erect and reach orgasm. Not as easily aroused*” (19 M) and “*I had to quit pornography to be able to orgasm and get hard*” (29 M). Others also cited diminished sexual functioning for pornography-assisted masturbation, such as: “*I find it increasingly more difficult to get aroused, even with pornography”* (26 M)*.* These respondents typically also indicated difficulties achieving arousal for offline masturbation, again suggesting a sexual reliance on Internet pornography.

##### Subtheme 2.2.3: orgasm function/satisfaction

Participants also connected their heavy pornography use with reduced orgasm functioning and sexual satisfaction with real partners. For instance, respondents remarked that their pornography use “*makes it harder to reach orgasm without porn*” (21 M) and that “*porn has made it harder to get erect with real women, and impossible to orgasm”* (37 M). Some also reported diminished sexual satisfaction while using pornography, such that *“my sensitivity is diminished and the activity is more habitual than satisfying”* (26 M; also see subtheme 4.2.1 regarding diminished pleasure).

### Theme 3: post-pornography complaints

Respondents reported a range of perceived acute adverse consequences following pornography use. These were broadly categorised as cognitive deficits (such as concentration difficulties), depressive emotional and physical complaints, and elevated social anxiety, suggesting immediate negative effects on individuals' mental and physical health.

#### Subtheme 3.1: cognitive experiences

Some participants reported notable cognitive deficits shortly after using pornography, generally summarised as ‘brain fog’. As one man stated, “*when I am frequently masturbating with porn, I find my mind becomes foggier”* (26 M). Another described this brain fog as *“feeling "dumb," maybe a little "wired””*, which he directly linked to certain intensity indicators like edging and binges: *"What I think is happening, is I jump between different pictures/videos/games (while "edging") which causes the "time jumps." Then, since I lose track of time, I spend an unhealthy amount of time doing this, leading to brain fog afterwards"* (27 M). Respondents did not generally report similar problems following engagement in other sexual behaviours.

#### Subtheme 3.2: anhedonia, fatigue and amotivation

Respondents also described heightened depressive symptoms following pornography use, especially unexplained physical and mental lethargy and amotivation for everyday tasks. For instance, one respondent remarked that “*After using porn, my energy and motivation drops dramatically. Tasks I'd had planned on doing, even as simple as cleaning your bedroom feel huge jobs*” (18 M) and “*I feel as if my reward system has been completely drained, and completing tasks afterwards becomes very challenging”* (23 M). Some also linked these complaints to the subjective intensity of the sexual experience, for instance: “*The longer and more intense the session, the greater the mental exhaustion*” (25 M). Similar issues did not generally result from other sexual behaviours (or were notably less severe than following pornography use). However, there was some evidence for a role of third variables like PPU-related distress and sleep loss from excessive pornography use.

#### Subtheme 3.3: social anxiety

Respondents also described elevated social anxiety following pornography use. This was typically described alongside other mental and physical effects, such as “*Less energy and motivation, brain fog, social anxiety*” (27 M) and “*Depression, lack of motivation, social anxiety, social isolation*” (20 M), and numerous respondents called for increased therapist awareness of such issues. Some individuals also indicated heightened anxiety following other sexual activities, albeit to a lesser degree.

### Theme 4: altered state of sexual arousal

Respondents also reported subjective changes to their masturbatory experience when using Internet pornography. Here, two distinct patterns emerged: reduced pleasure during the masturbatory experience, and enhanced mental sensations (relative to physical stimulation) during the experience.

#### Subtheme 4.1: diminished sensitivity or pleasure

Some respondents reported diminished pleasure from pornography-assisted masturbation, broadly reflecting desensitisation or habituation effects. This was often summarised as diminished genital sensitivity, such as taking “*a long time to orgasm. Not fully erect. Less sensitivity than before use*" (19 M). Others similarly suggested that both physical and mental sensitivity had waned over time: “*The lackluster feeling [is] mental and physical. After each session, it is like I slowly need more and more of it. Something new or fresh*” (22 M). Despite this diminished sensitivity, respondents still felt compelled to continue the behaviour, describing “*a strong compulsion or fascination to view porn. Actual sensation of masturbating has a numb feeling”* (23 M).

#### Subtheme 4.2: enhanced mental sensations

Some respondents also indicated a discrepancy between the mental and physical sensations that arise while consuming pornography, whereby the mental stimulation became the focal point of the masturbatory experience. For instance, one man remarked that “*pornography usually produces a powerful adrenaline response. The mental arousal is far stronger, the physical sensations less so*” (26 M), while another respondent described a trance-like state seemingly characterised by mental fixation: “*Time passes quickly, probably similar to someone in a casino… I would describe the mental effect to be similar of being engrossed in something to the point of losing track of time… The physical sensations are hard to describe since I don't have strong memories of them*” (27 M).

Some also suggested a connection between these enhanced mental sensations and the post-pornography affective and cognitive complaints described in Theme 3. For instance, one man remarked that when using pornography “*I feel a burst of chemicals within my brain. It feels a lot better, but is also quite draining and leaves me feeling spent”* (25 M) which aligned with similar testimonies like: “*I feel as if my reward system has been completely drained, and completing tasks afterwards becomes very challenging*” (23 M).

### Theme 5: intensity indicators

Respondents described certain usage patterns or strategies reflecting an increasing intensity of pornography consumption. These included increases in the amount of pornography use and/or content intensity, frequently moving between different stimuli, and pornographic binges that sometimes included delaying orgasm to prolong sexual gratification. These intensity indicators were often described alongside perceived adverse after-effects, sexual functioning problems, and subjective changes to the sexual experience with pornography and were therefore included as an integrative theme.

#### Subtheme 5.1: qualitative and quantitative escalation

Participants commonly indicated a need for greater stimulation over time, citing increasing time spent with pornography and/or greater stimulus intensity. This was typically a compensatory strategy to offset diminished sensitivity, captured by such sentiments as "*I have gradually sought more and more depraved material over time. Although very tame pictures would suffice in the past, I now seek out many different types of genres which could be considered extreme*" (27 M). Contrarily, there was little anecdotal evidence that these escalating usage patterns were driven by sexual exploration and discovery.

#### Subtheme 5.2: sexual novelty including tab-jumping

Many respondents indicated frequently moving between stimuli within a sitting, typically to heighten/maintain arousal: “*Frequent novelty is important. I cannot reach the highest highs without periodically changing the pornography*” (25 M). Others emphasised that tab-jumping was motivated by finding the ideal stimulus, leading the user to “*frequently move between different content to find the "best" one. But I'm never satisfied. The search can be hours long*” (26 M). Indeed, these respondents typically described escalating use, including pornographic binges. Collectively, these reports indicate that some users employ tab-jumping as a compensatory strategy to overcome desensitisation effects, especially as part of pornographic binges (i.e., edging), as described further in the next sub-theme.

#### Subtheme 5.3: binges and edging

Some respondents also reported pornographic binges, often entailing hours-long sessions, sometimes with repeated orgasms. These binges were also characterised by reduced sensitivity over the course of the session, as well as delaying climax while moving between many stimuli (edging), reflected in such testimonies as “*when* [my partner’s] *out shopping on a weekend I can edge for hours*” (30 M), and *“I orgasmed 12 times in a day, sensitivity diminished each time, but porn kept me going”* (27 M). Respondents also described that these periods of heavy use were motivated by a pursuit for ever-new and exciting stimuli, such as "*I've spent hours searching for new material before. I've also tried to "max out" the number of orgasms within a day*" (35 M).

Respondents also linked these binge behaviours to the various under-studied domains described above. One man clearly cited an altered state of sexual arousal when describing the binge experience, stating that “*the physical sensitivity reduces as time goes on, but the mental arousal is still there*” (26 M). Others described a hangover-like effect following pornographic binges, such as feeling “*mentally and physically exhausted. I’m drained from a session*” (25 M), and “*general lethargy after binge*” (19 M). Binges also induced sexual exhaustion, which was subsequently linked with prolonged sexual functioning concerns: “*I suffer from impotence for days after extended masturbation. I feel 0 attraction whatsoever towards my partner after I binge pornography. I couldn’t get an erection from my partner even if they held a gun to my head*” (25 M).

## Discussion

This study aimed to provide a nuanced understanding of the experiences of individuals with self-identified PPU, focusing on various under-studied features. We conducted a thorough investigation of dimensions that have received only preliminary investigation, including perceived adverse after-effects, pornography-related sexual dysfunction, and subjective changes to the consummatory experience when using pornography. Additionally, we targeted features related to inner conflict and distress given recent calls for greater understanding of these dimensions^23,24^. Our rich qualitative investigation, which involved an online sample of people who self-identified as experiencing PPU, generated new insights that can inform subsequent research and clinical practice. In the following sections, we discuss these insights and their implications in greater detail.

### Disentangling psychological conflict in PPU

Recent developments highlight the need to better understand the ways in which people who identify as having PPU experience distress and how inner conflict relates to their pornography use^21,24^. One key psychological model for investigating inner conflict and distress in PPU is moral incongruence^20,26^. Although researchers initially assumed that moral incongruence stems primarily from religious or conservative attitudes that forbid or discourage pornography use^22^, recent work challenges this narrow conceptualisation by demonstrating that users can have moral objections to their pornography use for many other reasons^21,24^. Firstly, our findings illustrate how addiction-related distress, which may manifest as feelings of shame or guilt over a lack of behavioural control despite negative consequences, can be seen as a type of moral incongruence^7,46^. Wright^23^ has argued that harmful behaviours are perceived as inherently immoral, and this perception can contribute to feelings of moral conflict in individuals who struggle with addiction-related distress.

Similarly, individuals who consume pornographic genres that depict socially unacceptable activity, such as violent or non-consensual acts, may experience distress when they encounter content that conflicts with social norms and their personal values and beliefs^6^. As these sources of inner conflict are not necessarily linked to religious or conservative views, this challenges previous conceptualisations that moral incongruence is primarily driven by prohibitive attitudes toward pornography use in general. These findings highlight the need for further research toward the complexities of moral incongruence and to distinguish it from other psychological processes relevant to PPU, which will assist researchers and clinicians to develop effective and targeted interventions^21,46,47^. Implications regarding conceptualisation are elaborated below.

### Under-explored functional impairments

Our findings shed new light on various sexual and non-sexual functional impairments related to PPU that have yet to be robustly examined in the existing literature. Firstly, our study supports a link between PPU and sexual functioning deficits, which is broadly consistent with clinical case reports,^12,13^ qualitative research,^6–8^ and emerging survey data^18,48,49^ (although discrepant findings are also noted^50^). However, the issue appears complex, with many respondents reporting varying combinations of negative effects, including decreased libido, sexual arousal, and orgasm function with real partners. Methodologically, our study recommends differentiating between specific sexual behaviours when measuring sexual functioning^9,51^ and directly quantifying pornography-related sexual functioning concerns^52^. Increasingly complex models that control for third variables such as depression and anxiety, offline sexual history, and clinically relevant intensity indicators like pornographic binges are also required^28,29,53^. Furthermore, longitudinal and intervention data are necessary to address uncertainties and improve clinical practice^16,20^.

Secondly, our findings are consistent with other exploratory studies that suggest some individuals with PPU describe various cognitive, affective, social, and physical complaints soon after intense periods of pornography use^6,7,9,10^. Like sexual functioning concerns, these complaints are not universal and appear limited to heavy chronic pornography users, and especially pornographic binges^29^. Future work clarifying the dimensions, neurobiological substrates, and potential mechanisms behind such complaints would be informative.

### Emphasising the qualitative aspects to pornography use

Our findings also demonstrate the need for researchers and clinicians to look beyond the mere quantity of pornography use in the context of PPU. In particular, our work provides useful insights regarding intensified patterns of pornography use such as tolerance/escalation and pornographic binges. Although some scholars have expressed doubt as to the legitimacy of tolerance/escalation in CSBD^54^ and behavioural addictions more broadly^55^, our findings corroborate increasing evidence that many individuals with PPU experience tolerance and desensitisation effects, which can lead to escalating use^25,56^. This supports the addiction conceptualisation for at least some individuals with PPU. Our findings also suggest that pornographic binges, which often involve tab-jumping, viewing compilation videos, and edging, constitute another dimension of escalating use that warrants further assessment^29^. Binge behaviours are increasingly recognised in other behavioural addictions, although investigation in PPU remains scarce^29,33^. Our findings also tentatively link pornographic binges with perceived adverse after-effects, a subjectively altered state of sexual arousal, and pornography-related sexual functioning concerns, at least in some heavy pornography users. As such, research assessing the relationships between increasingly heavy use and a host of PPU-related dimensions is warranted^6,7,29^.

To our knowledge, this study is also the first to directly investigate subjective changes to consummatory experience during pornography use, broadly reflected by changes to the mental and physical stimulation that arises while masturbating. We identified two potential forms of subjective changes: one characterised by diminished pleasure (which is a symptom recognised under CSBD in ICD-11 based largely on cue reactivity data^57^) and another in which mental stimulation becomes increasingly important as the user engages in frequent novelty-seeking or binges. Temporal dissociation and a trance-like state, as cited in the Internet addiction literature^15,58,59^, may also be relevant to these subjective changes.

### Conceptualisation and theoretical specificity

Although PPU is traditionally considered a sub-type of CSBD^27,47,60^, recent work challenges this orthodoxy, suggesting that PPU may constitute a distinct entity characterised by dysregulated sexual activity specifically related to pornography use^3,61^. This suggests that PPU is not simply a consequence of general hypersexuality, but is driven by unique underlying mechanisms, including the structural features of Internet pornography that potentially accelerate addiction-related psychological and appetitive mechanisms^3,4,62^. Indeed, we found no significant evidence that individuals with PPU experience similar dysregulated sexual desires or behaviours for other sexual activities. Instead, PPU appears better characterised by specific usage patterns related to pornographic binges and increasing tolerance and escalation in pornographic use^29,63^. We also identified several features of PPU that are not fully accounted for by existing theoretical frameworks, including pornography-related sexual dysfunction and a subjectively altered sexual experience while engaging with pornography. As described above, we also found that inner conflict and distress can take various forms that are largely unique to PPU relative to other forms of CSBD^24^. Overall, these findings highlight the need for further research to better understand the distinct features and potential subtypes of PPU and their underlying mechanisms.

To advance our understanding of PPU, future research should investigate the similarities and differences in aetiology and associated features between PPU and other potential behavioural addictions, including CSBD^4,61^. This inquiry is necessary to promote clarity regarding the conceptualisation, diagnosis, and treatment approaches for PPU^28,64^. Furthermore, studies should continue to explore potential subtypes of PPU that align with various conceptual models, rather than seeking a one-size-fits-all approach.^28,65,66^.

### Strengths and limitations

Our research design yielded several notable benefits. Foremost, our qualitative design allowed respondents to describe the PPU lived experience in their own words. Recruiting respondents from various online sources also allowed us to engage individuals across a wide range of PPU severity. Moreover, within-case analysis enhanced our understanding by triangulating information across numerous relevant dimensions, while follow-up questions permitted us to clarify initial impressions.

Several limitations are also noted, most notably that our findings are not intended to be generalisable given the qualitative design. Our online survey also likely offered less informational power than semi-structured interviews, especially for issues that may be difficult to describe, including subjective changes to the sexual experience; as such, other peripheral features of PPU may exist that we did not identify. As with much of the PPU literature, our sample also lacked sexual and cultural diversity^67,68^. Our cross-sectional data also do not allow for causal inference, which requires longitudinal and intervention data. Examining the effects of prolonged abstinence following chronic and heavy use is an avenue for future work, as is reconditioning the masturbatory response without pornography (broadly akin to a tapering schedule in substance abuse recovery)^12,69^. Finally, given our online sample of individuals self-identifying as experiencing PPU (engaged through various social media platforms), we cannot assume that these findings would generalise to those who use pornography per se.

## Conclusion

Our study contributes to the growing body of literature on problematic pornography use by shedding light on some of its lesser-known and understudied features (namely, cognitive, affective, social, and physical complaints following intense periods of pornography use, sexual functioning problems with real partners, and subjectively altered arousal states while using pornography). Importantly, it seems that not all individuals with PPU experience these specific characteristics, and future work should interrogate their dimensions, relative prevalence, and underlying mechanisms to enhance our understanding of this complex phenomenon. Although current theoretical models for PPU have been useful in understanding its common characteristics^4^, they may not fully capture its multifaceted and unique features^28,70^. Further research investigating these nuances and complexities is required to ultimately enhance research and clinical outcomes related to PPU^6,70^.

### Supplementary Information

Supplementary Information.

## Data Availability

The datasets generated during and/or analysed during the current study are not publicly available due to ethics approval conditions set by the Monash University Human Research Ethics Committee, but are available from the corresponding author on reasonable request.
