# Supplementary material for: Clarifying and extending our understanding of problematic pornography use through descriptions of the lived experience
Source: Sci Rep. 2023 Oct 24;13:18193. doi: 10.1038/s41598-023-45459-8 (PMC10598215; doi:10.1038/s41598-023-45459-8)
Supplement: Supplementary file 1 — Supplementary Information. [file 41598_2023_45459_MOESM1_ESM.docx]

## File S1: Supplementary materials - Qualitative survey and demographics

**Socio-demographics:**

- Country of residence
- Age
- Sex assigned at birth & gender identity
- Sexual orientation
- Employment status
- Highest level of completed education
- Relationship status
- Level of religiosity [0-4 Likert scale]

**Distress/inner conflict**

- **Distress from pornography use**: “*Have you ever felt distress about your pornography use? This might relate to physical health, mental/emotional health, social interactions/relationships, sexual functioning, moral/spiritual/religious beliefs. If so, please describe.”*
- **Moral incongruence**: “*Do you believe that using pornography is morally or ethically wrong? Why/why not?*”

**Perceived adverse after-effects following pornography use**:

- **After masturbating to pornography**: “*Have you ever experienced negative unintended effects in the hours after using pornography? (e.g., related to mood/mental functioning, socialising, how you feel physically, or sexual functioning)*” [yes/no]
  - Those who answered affirmatively were then asked: “*Please describe these negative/unintended experiences. This might include mental, physical, emotional, or social aspects*”, “*What do you think explains these experiences?*”, and “*Have you experienced similar negative effects after other sexual activities (e.g., partnered sex, porn-free masturbation, etc.)*?”

**Sex life characteristics**

- **Perceived impact of pornography use on sexual functioning**: “*Here, sexual functioning relates to things like your sensitivity to touch, how easily you're aroused, or orgasm satisfaction. Would you say that your pornography use has impacted your sexual functioning, either positively or negatively? If so, please describe*.”
- **Sexual functioning for different sexual behaviours:** *Please describe your sexual functioning in the following situations* [separate free-text inputs]. Note: Please tell us if you haven't tried any of these behaviours in the past six months, but then describe how you think you'd respond if you were to try: i) pornography-assisted masturbation, ii) masturbation without pornography; iii) sex with a partner.
- **Sexual appetite for pornography vs. offline behaviours**: “*How strong is your sexual appetite for pornography compared to other sexual activities (e.g., partnered sex, porn-free masturbation)?*” and “*When faced with an urge, how interested would you be to have sex with a partner or masturbate without pornography instead?*”

**Consummatory experience associated with pornography use**:

- **Using pornography vs. other sexual behaviours**: “*Here we want to know how the sexual sensations (i.e., the mental/physical feelings) that you get from using pornography compared to those from other sexual behaviours. Please rate the following statement: When I use pornography, I experience different sensations that I don't get from other sexual activities (e.g., compared to porn-free masturbation or partnered sex)”* [1= Strongly disagree, 5 = Strongly agree]
  - Those who responded affirmatively (rating 4 or 5) were then asked: “*In what ways does pornography feel different compared to other sexual behaviours? (e.g., partnered sex/porn-free masturbation). Here, you might consider certain mental and/or physical sensations that arise during, before, or after these sexual behaviours. [altered sexual experience]*”
- Tab-jumping tendencies (*How important is sexual novelty when you masturbate to pornography? For example, are you sufficiently aroused by a few images/videos, or do you move frequently between tabs?*).

**Intensity indicators**:

- **Pornography usage per week**: “*Approximately how many hours per week do you currently spend watching or searching for pornography?”*
- **Binges: “***Here, we define pornographic binges as masturbating to pornography for several hours straight or for a significant greater number of times in a day than normal. Have you ever 'binged' with pornography?*” [yes/no]
  - Those who responded affirmatively then responded to the following questions: “*Please describe such binge experiences. You might consider the following aspects: frequency/duration of binges, number of orgasms, your sensitivity across the session, etc.*”, “*How do you typically feel in the hours after a binge? This might relate to your physical, sexual, mental, emotional, and/or social functioning. Relatedly, how influential are other factors like frustration, loss of sleep, not having eaten food/drunk water, or similar?*”
- **Escalating use**: “*Have your patterns of pornography use changed over time? If so, please describe.*”
- **Role of kinks/fetishes toward one’s pornography use**: “*Have any kinks/fetishes played a notable role in your pornography use? If so, how? (But do not describe specific kinks/fetishes - just the general ways that these relate to your porn use).*”
- **Importance of sexual novelty (including tab-jumping)** “*How important is sexual novelty when you masturbate to pornography? For example, are you sufficiently aroused by a few images/videos, or do you move frequently between tabs?*)”

**Other features**:

- **Clinical characteristics relevant to seeking treatment**: “*What kinds of issues or symptoms would you like therapists or clinicians to acknowledge when assessing problematic pornography use?*”

**Attention checks (embedded throughout survey):**

To show that you're paying attention, please leave the following text box blank

|  |
| --- |

To show that you're paying attention, please select the option "Piano keys"

- Royal blue
- Violet flower
- Finger
- Piano keys

## Table S1. Illustrative quotes

| **Themes, subthemes, and illustrative comments** | **Age, sex** | **Recruitment stream** |
| --- | --- | --- |
| ***Theme 1: Internal conflict*** |  |  |
| 1.1: Conflict from diminished control |  |  |
| - ***Emotional, feeling sad, angry, and annoyed that I caved in again****… I felt like I was consumed by porn and unable to stop it, that I was an addict.”* | 22M | Social media |
| - “***Shame and disappointment in self*… *from multiple attempts to quit using porn***.” | 23M | PPU forum |
| - “*My mental wellbeing is worse and* ***I feel depressed about my porn usage. I feel like what I am doing is hindering me in life*.”** | 26M | PPU forum |
| - “*I am* ***ashamed. I wish I could quit so I could function normally*** *in a relationship.* ***Worried I will never quit****.”* | 22M | PPU forum |
| - “***Feeling extremely guilty, feeling shame… It's a horrible, horrible mental addiction*** *that can destroy people and impact them much more than they realise*” | 29M | PPU forum |
| - “*Post nut clarity is the best term used to describe the hours after watching porn. You feel a sense of* ***guilt and annoyance that your desires control you***.” | 21M | Social media |
| - “***Lots of shame & embarrassed that I've returned to porn***." | 23M | Social media |
| - "*I feel like* ***my addiction has prevented me from being myself and making meaningful bonds****.”* | 18M | Social media |
| 1.2: Conflict over the genres consumed |  |  |
| - “*Many pornography addicts have* ***seen things they regret, myself included****. Things that scar your brain and* ***traumatize you forever****.*” | 25M | PPU forum |
| - “*Things have* ***escalated to disturbing, unethical and troubling levels****. Even just that has caused (and will cause until resolved) a* ***very high level of distress***." | 29M | PPU forum |
| - “*The content of the pornography, which is often very* ***violent and misogynistic*** ***makes me feel - rightly - like shit*.**” | 24F | Social media |
| - “*My primary porn consumption involves kinks and fetishes. Over time, these have changed and become more extreme. Again, there is nothing illegal, but* ***the content elicits more shame and guilt***.” | 26M | PPU forum |
| - “*I used to masturbate to nonextreme stuff* [sic] *but it escalated as I got older…* [my use of kink/fetish pornography] ***made me question my gender and sexuality***.” | 22M | PPU forum |
| - “*Definitely* [having consumed] ***stuff that tends towards violence misogyny and degradation***… [I’m] *worried about sexualising vulnerability in women and aggression in men*.” | 24F | Social media |
| 1.3: Pornography use exacerbating underlying issues/attitudes |  |  |
| - *The use of pornography* ***exacerbates all other problems****.*" | 28M | Social media |
| - “[*Using pornography is a*] ***compensation for loneliness and depression***." | 43M | PPU forum |
| - “*I also feels it [using pornography]* ***adds to my anxiety and depression***." | 26M | PPU forum |
| - “*Feeling of* ***unsatisfaction*** *[sic]* ***because I wasn't with someone****. Also due to the fact that* [sic] *I am* ***still single***.” | 20M | Social media |
| - “*It made me feel shameful and guilty…. I view it [pornography use] as close to* ***cheating on your partner***.” | 21M | PPU forum |
| - “*I feel gross afterwards usually for the rest of the day or into the next morning.* ***I feel like I've done something horribly wrong*.**” | 22F | Social media |
| - “*Guilty or gross with yourself for wasting time and* ***feeling like a dirty person***.” | 23F | Social media |
| - “*[After using porn I feel]* ***Really guilty, dirty, I feel like I need a shower*** *and even after shower I don't feel good”* | Age_NS_F | Social media |
| ***Theme 2: Impact on sex life*** |  |  |
| 2.1: Decreased sexual intimacy and enjoyment |  |  |
| - "*Negative impact [of pornography on sexual functioning] -* ***feel less connected when sexually intimate with real partner***." | 23F | Social media |
| - "*I feel porn has distorted my perception on sex, as well as* ***impacting my expectations on sharing an intimate exchange***." | 23M | Social media |
| - “*Negatively [impacted by pornography use]. It has been* ***difficult to enjoy sex with a real partner*** *despite being aroused and having no physical problems*.” | 35M | PPU forum |
| - “***Less in touch with partner***.” | 56M | PPU forum |
| - “*In periods of time when my watching of porn increases,* ***sexual interaction with my partner* decreases.**" | 21NC | Social media |
| - "*It probably has impacted it negatively, in that I have a* ***misguided vision of healthy sexual activity*.**" | 24M | Social media |
| - “*It has* ***created unrealistic expectations of intimacy with my partner*** *and also leads to orgasm sooner*.” | 22M | Social media |
| - "*I haven't had sex before but I think viewing pornography has given me* ***unrealistic expectations of how it really is***." | Age_NS_F | Social media |
| 2.2 Perceived adverse effects on sexual drive and arousal |  |  |
| 2.2.1 *Sexual desire/motivation* |  |  |
| - *“Negatively* [impacted]*. It* ***makes me not motivated to go out and pursue sexual relationships with a partner****. If I abstain for a long time from pornography I feel more motivation/need to pursue a partner.”* | 25M | Social media |
| - “*It [pornography] has* ***lowered my sex drive in the past to where I would have rather masturbated to porn*** *sometimes or I wouldn't initiate sexual encounters with my partner*.” | 34M | PPU forum |
| - "*It [pornography]* ***makes me not motivated to go out and pursue sexual relationships*** *with a partner*.” | 25M | Social media |
| - “*Struggle for erection and interest,* ***lower libido***." | 26M | PPU forum |
| - “*I enjoy sex but often* ***don’t have much of a libido***.” | 25M | PPU forum |
| - “*Guess it makes me* ***less likely to initiate sex***.” | 30M | PPU forum |
| - "*I think it* ***makes me comfortable not putting myself out there to find a partner*** *if I know I can pleasure myself sexually with porn*." | 20NC | Social media |
| - “*After using porn, my energy and motivation drops dramatically…* ***my attraction to my partner significantly drops***.” | 18M | Social media |
| 2.2.2 *Sexual arousal* |  |  |
| - "*Negatively* [impacted by pornography use], ***harder to orgasm and harder to stay hard***." | 23M | Social media |
| - "*I think it has* ***100% impacted my sexual functioning, both in arousal and performance***." | 18M | Social media |
| - “*I believe porn has* ***made it harder to get erect with real women****, and impossible to orgasm*.” | 37M | PPU forum |
| - “*Negatively* [impacted by pornography use]*. I find it* ***increasingly more difficult to get aroused, even with pornography*.”** | 26M | PPU forum |
| - *“Negatively* [impacted by pornography use]*. When I watch porn often my* ***sexual functioning seems to decrease*.**” | 20M | PPU forum |
| - “*Negatively* [impacted by pornography use]*,* ***reduced sensitivity and more difficult to get erect and reach orgasm. Not as easily aroused****.”* | 19M | PPU forum |
| - “*I* ***had to quit pornography to be able to orgasm and get hard****.”* | 29M | PPU forum |
| - “*Porn induced* ***erectile dysfunction, delayed orgasms, and lack of overall penile sensitivity***.” | 27M | PPU forum |
| - “***Struggle for erection*** *and interest, lower libido."* | 26M | PPU forum |
| - “*Extremely negatively* [impacted by pornography use]. *I have* ***frequently faced issues with impotence since I was 16 years old***.” | 25M | PPU forum |
| - “*Negative* [impact of porn on sexual functioning]. ***I am unable to function***.” | 22M | PPU forum |
| - “*Not sure to be honest because I haven't any sexual experiences with a partner but* ***I'm worried that my sexual functioning has been negatively impacted***” | 20M | Social media |
| 2.2.3 *Orgasm function & satisfaction* |  |  |
| - “*It* ***makes it harder to reach orgasm without porn***.” | 21M | Social media |
| - “*I believe* ***porn has made it harder to get erect with real women, and impossible to* *orgasm***.” | 37M | PPU forum |
| - “*Negatively impacted sexual functioning.* ***I can only orgasm when viewing pornographic material*** *and cannot orgasm when stimulated by a partner*.” | 18NC |  |
| - “*Negatively,* ***reduced sensitivity and more difficult to get erect and reach orgasm****. Not as easily aroused*.” | 19M | PPU forum |
| - “***Delayed ejaculation***.” | 38M | PPU forum |
| - “***Takes a long time to cum when having sex***. | 25M | Social media |
| - “***Increased time to orgasm. Less in touch with partner***.” | 56M | PPU forum |
| - "*Sometimes negatively as* ***I might not be able to finish as quickly with a partner***." | 22M | Social media |
| - "*Yes, find it* ***difficult to orgasm without the mental / visual competent [sic] of porn****. Regular masturbation with the aid of porn seems much more satisfying that the attempts of a sexual partner*." | 23F | Social media |
| - "*Definitely negatively* [impacted by pornography use], *masturbating to pornography* ***makes me less energetic and sensitive, and enjoy sex less*.**" | 27M | Social media |
| - “*I* ***have to imagine or think of porn in order to reach satisfaction***." | 22F | Social media |
| ***Theme 3: Perceived adverse after-effects*** |  |  |
| 3.1 Cognitive experiences |  |  |
| - “*When I am frequently masturbating with porn, I find my* ***mind becomes foggier.***” | 26M | PPU forum |
| - “[After using pornography I feel] ***Drained, distracted****, jumpy*.” | 25M | PPU forum |
| - “*Reduced desire to socialise, tiredness, feeling guilty, lack of motivation,* ***difficulty concentrating***.” | 19M | PPU forum |
| - “*Feeling "****dumb****," maybe a little "wired”… I spend an unhealthy amount of time doing this* [masturbating with pornography]***, leading to brain fog afterwards***.” | 27M | Social media |
| - “*I feel mental exhaustion* [after using pornography] *and shame. I also feel* ***mentally and physically exhausted****. I’m drained from a session."* | 25M | PPU forum |
| - “*Around 8 months ago, I decided to take a* ***porn-free journey to rid myself of it****, and the mental and physical positive affects* [sic] *are endless…* ***less brain fog****, better mood regulation, less stress, less irritability, more energy, more motivation, less shame, more confidence, less social anxiety, etc*.” | 27M | PPU forum |
| 3.2 Anhedonia, fatigue & amotivation |  |  |
| - **“*Less energy and motivation*** *[after using pornography], brain fog, social anxiety*.” | 27M | Social media |
| - “*I feel as if my reward system has been completely drained, and* ***completing tasks afterwards becomes very challenging***.” | 23M | Social media |
| - “*After using porn,* ***my energy and motivation drops dramatically****. Tasks I'd had planned on doing, even as simple as cleaning your bedroom feel huge jobs*.” | 18M | Social media |
| - “*It is something that changes based on the length of the session. The* ***longer and more intense the session, the greater the mental exhaustion***.” | 25M | PPU forum |
| - “*I feel* ***extremely tired mentally and emotionally.*** *I would become a recluse uninterested in anything (people, activities, etc.).”* | 22M | PPU forum |
| - *“Feeling of* ***depleted energy”*** | 22M | Social media |
| - *"Tired, depressed, anxious, shame… When I am frequently masturbating with porn, I find* ***my mind becomes foggier, I feel less confident/motivated****.* | 26M | PPU forum |
| - “*Reduced desire to socialise, tiredness, feeling guilty,* ***lack of motivation, difficulty concentrating****.”* | 19M | PPU forum |
| - “*I feel mental exhaustion* [after using pornography] *and shame. I also feel* ***mentally and physically exhausted****.* ***I’m drained from a session****."* | 25M | PPU forum |
| - “*Around 8 months ago, I decided to take a porn-free journey to rid myself of it, and the mental and physical positive affects* [sic] *are endless… less brain fog, better mood regulation, less stress, less irritability****, more energy, more motivation****, less shame, more confidence, less social anxiety, etc*.” | 27M | PPU forum |
| - “***Tired, not willing to do anything****. My day feels practically done even if I do it early in the morning I just, stop doing anything unless I have work. I hate it*." | 30M | PPU forum |
| - “*Don't feel like doing anything besides laying* [sic] *in bed… It would also sometimes make me* ***feel extremely tired mentally and emotionally****. I would become a recluse uninterested in anything (people, activities, etc.).”* | 22M | PPU forum |
| - "***Depressive symptoms, low motivation****, doom scrolling, social isolation*" | 23M | PPU forum |
| - “[Using pornography] ***drains energy in a negative way***.” | 23M | Social media |
| - “***Lack of energy and motivation to do anything***.” | 28M | Social media |
| - “***Low motivation and drive to do anything****. Don't feel like talking to anyone but it returns back to normal by an hour. But low motivation and drive lasts for the whole day if I watch porn & masterbate* [sic] *in the morning*.” | 22M | Social media |
| - “***I feel unmotivated for things after porn****. I feel down. It makes me want to skip on all social aspects even on ones I have planned. It affects my study too. It makes me unmotivated to meet deadlines for school/work. It makes me want to interact less with people especially my family*.” | 25M | Social media |
| 3.3 Social anxiety |  |  |
| - “*Less energy and motivation, brain fog,* ***social anxiety*.**” | 27M | Social media |
| - "***Socially anxious***." | 43M | PPU forum |
| - “***Hard to socialise after*** *with brain fog*.” | 23M | Social media |
| - “*Around 8 months ago, I decided to take a porn-free journey to rid myself of it, and the mental and physical positive affects* [sic] *are endless… less brain fog, better mood regulation, less stress, less irritability, more energy, more motivation, less shame, more confidence****, less social anxiety****, etc*.” | 27M | PPU forum |
| - “*Depression, lack of motivation,* ***social anxiety****, social isolation*.” | 20M | PPU forum |
| - “*Sexual dysfunctions caused by porn use,* ***social anxiety***.” | 19M | PPU forum |
| - “*No desire to socialize.* ***Horrible social anxiety****. Toxic self-shame*.” | 21M | PPU forum |
| - "*I feel as if porn* ***makes me more socially anxious****, and greatly* ***reduced my confidence in social situations****."* | 23M | Social media |
| ***Theme 4: Altered state of sexual arousal when using pornography*** |  |  |
| 4.1 Diminished sensitivity/pleasure |  |  |
| - “*A strong compulsion or fascination to view porn.* ***Actual sensation of masturbating has a numb feeling***.” | 23M | PPU forum |
| - “[Pornography-assisted masturbation] *can take a long time to orgasm.* ***Not fully erect****.* ***Less sensitivity than before use***." | 19M | PPU forum |
| - “*I find it increasingly more difficult to get aroused, even with pornography.* *My* ***sensitivity is diminished and the activity is more habitual than satisfying***” | 26M | PPU forum |
| - “*Aroused by the thought of watching porn but it quickly declined while I was watching porn and* ***by the time I climaxed the excitement was gone****.”* | 22M | PPU forum |
| - “*The* ***lackluster feeling*** [when using pornography] ***is mental and physical****. After each session, it is like I slowly need more and more of it. Something new or fresh*.” | 22M | PPU forum |
| - “*The strength of both physical & mental sensations is generally quite high initially. When I start the "session" I'm usually excited and ready to go. However, if it lasts for more than 10-15 minutes,* ***the initial novelty wears off and I begin to just "go through the motions"*** *until it is done*.” | 26M | PPU forum |
| - “*I was aroused by the thought of watching porn but it quickly declined while I was watching porn and* ***by the time I climaxed the excitement*** ***was gone***.” | 22M | PPU forum |
| - “*The time and amount of stimulation would increase as the* ***mental and physical sensitivity decreased***.” | 27M | PPU forum |
| - "***Genitals are not particularly sensitive*** *(must be harsh in order to orgasm)*.” | 18NC | PPU forum |
| 4.2 Enhanced mental sensations |  |  |
| - *“Pornography feels better than sex. I try to masturbate for hours so I don't have to stop using pornography...* ***I feel a burst of chemicals within my brain****. It feels a lot better, but is also quite draining and leaves me feeling spent and ashamed.”* | 25M | PPU forum |
| - "*Pornography usually* ***produces a powerful adrenaline response. The mental arousal is far stronger****, the physical sensations less so*.” | 26M | PPU forum |
| - "*Porn kinda* [sic] ***feels like it’s melting my brain****. Gives me a high from over arousal*" | 30M | PPU forum |
| - "***Time passes quickly, probably similar to someone in a casino****. I'll often feel kind of "dumb" after, but also more satisfied… I would describe the mental effect to be similar of being* ***engrossed in something to the point of losing track of time*** *(like while doing a hobby or something) but more...horny.* ***The physical sensations are hard to describe since I don't have strong memories of them***." | 27M | Social media |
| - “*It's a* ***rush of brain chemicals and release***… *I usually* ***have to wind down my brain. It feels very stimulated***” | 43M | PPU forum |
| ***Theme 5: Intensity indicators (integrative theme)*** |  |  |
| 5.1 Qualitative & quantitative escalation |  |  |
| - “*I have found that* ***my taste has become more extreme, entering into fetishes I had found distasteful previously***." | 32M | Social media |
| - “*The lackluster feeling [is both] mental and physical. After each session, it is like* ***I slowly need more and more of it. Something new or fresh****.”* | 22M | PPU forum |
| - “*The* ***time and amount of stimulation would increase*** *as the mental and physical sensitivity decreased.”* | 27M | PPU forum |
| - "*I have gradually sought more and more depraved material over time.* ***Although very tame pictures would suffice in the past, I now seek out many different types of genres which could be considered extreme***." | 27M | Social media |
| - "*My* ***tastes have changed*** *and my* ***time spent indulging has increased****. I have* ***looked at things that I would have once found disgusting***." | 25M | PPU forum |
| - "***Time spent has generally increased***." | 26M | PPU forum |
| - *I need* ***more extreme content to feel satisfied****. Nothing illegal, but more "kinky" unorthodox content…* ***Over time, these have changed and become more extreme***." | 26M | PPU forum |
| - "***Hard to orgasm unless extreme***… [although] *It wasn't always the case.* ***I used to masturbate to nonextreme stuff but it escalated as I got older***." | 22M | PPU forum |
| - "***Time spent has increased significantly and I*** ***now use more extreme content***." | 19M | PPU forum |
| - “*I* ***use it more and seek more variety***." | 22M | PPU forum |
| - "*I keep* ***needing more and more***." | 32M | PPU forum |
| - "***Novelty has lead me down some deep rabbit holes****… I've definitely explored some things out of the normal realms of porn*." | 54M | PPU forum |
| - “*As my addiction got stronger I would go to more hardcore fetishes.*" | 27M | PPU forum |
| - “*The* ***time and amount of stimulation would increase as the mental and physical sensitivity decreased****… In an attempt to 'up the ante',* ***I would seek out things like that*** [kinks/fetishes] *to consume*." | 27M | PPU forum |
| - “*Things have* ***escalated to disturbing, unethical and troubling levels***… *The escalation often seen in addicts crosses many thick ethical red lines*." | 29M | PPU forum |
| - "*I frequently switch tabs,* ***often increasing the extremeness of the pornography****. I can have up to 5 tabs, sometimes*." | 18M | PPU forum |
| - "*I would say that* ***I just needed more porn and more specific categories****. At one point, I started even using VR porn and this changed my entire taste for pornography."* | 21M | PPU forum |
| - "***Over time the intensity of what I watch has slowly but surely increased***." | 20NC | Social media |
| - "*I definitely feel that* ***the time spent watching porn has increased over the years, as well as the level of stimulation has greatly increased also****… The* ***categories and fetishes have become more extreme*** *as the years have progressed of me watching this content.* | 23M | Social media |
| - “*I* ***use it more often*** *and don't get aroused as easily with it as I used to*." | 18M | Social media |
| - "***Time spent and stimulation needed has increased over past few years*** *despite entering a relationship*." | 22M | Social media |
| - "***As I have become desensitised to porn****, I become more partial to kinks/fetishes rather than "vanilla" porn as I find it boring. Sometimes these kinks and fetishes very -* ***what I find attractive one day may be repulsive the next or as soon as I reach climax*** *and no longer have an urge*." | 21NC | Social media |
| - “*Very basic porn can feel stale and doesn't trigger the brain so much anymore. Kinks/ fetish or unusual situations are sometime necessary for the brain to see something novel and break the boredom*." | 23F | Social media |
| - "***Kinks and or fetishes have changed my taste in porn and I can only get off to that*."** | 26M | PPU forum |
| - `"***The time spent has become shorter, but I would need more stimulation, more intense videos***." | 22M | Social media |
| 5.2 Tab-jumping |  |  |
| - “***Very frequently move between tabs****, perhaps view snippets in excess of 100 videos or more*.” | 27M | Social media |
| - “[Frequently changing stimuli is] ***very important****. It's like I keep needing to know what the next image/video will be*.” | 27M | Social media |
| - “*Novelty is important…* ***I frequently move between different content to find the "best" one****. But I'm never satisfied. The search can be hours long.”* | 26M | PPU forum |
| - “***Frequent novelty is important****.* ***I cannot reach the highest highs without periodically changing the pornography***.” | 25M | PPU forum |
| - "***I frequently switch tabs****, often increasing the extremeness of the pornography.* ***I can have up to 5 tabs, sometimes***." | 18M | PPU forum |
| - "*I would search for the next video before the current one had even reached halfway.. Before the sex was even done on this video,* ***I'd be clicking on the next (or I'd skip to the orgasm part and then click the next). I'd say novelty was a very important factor, as an addict***." | 22M | PPU forum |
| - "*I think* ***I definitely move frequently between tabs****. I rarely masturbate to the same video back to back*." | 20M | PPU forum |
| - "***I'll have so many tabs*** *that the resize to fit them all makes it* ***near impossible to count***." | 30M | PPU forum |
| - "***I move frequently between tabs****. But only because the delayed orgasms I experience cause me to* ***seek multiple videos to get me through a 45 minute session****."* | 27M | PPU forum |
| - "***I frequently move between tabs****. Each new video/image I get even more aroused*." | 25M | Social media |
| - "***Definitely scroll between tabs***." | 23M | Social media |
| - "*Sometimes* ***I spend a very long period of time scrolling*** *through porn*." | 21M | Social media |
| - "***Frequently move*** [between content]" | 22M | Social media |
| - “*If I'm very aroused, a few images/videos will do but* ***normal takes a variety of tabs to finish***." | 20M | Social media |
| - *"****Frequently move between tabs****, but often rewatch the same videos*." | 23F | Social media |
| - "*I often* ***need to use lots of videos because I get bored really easily***." | 26F | Social media |
| - "***Frequently move across multiple tabs***." | 28M | Social media |
| - "***Very frequently changing content***." | 22M | Social media |
| - "*I* ***go between weeks of frequently moving between tabs****, and weeks of being fixated on one specific video/type of video*." | 22M | Social media |
| 5.3 Binges & edging |  |  |
| - "***I've spent hours searching for new material before****. I've* ***also tried to "max out" the number of orgasms*** *within a day*" | 35M |  |
| - "*When I can,* ***I will sit for up to 1.5 hours masturbating and "edging"*** *to many videos and GIFs until I find the "right" one. Right being subjective as I can deem it "unworthy" and* ***continue the search and prolong the pleasure****, while leading to a bigger climax*" | 26M | PPU forum |
| - "*When she's out shopping on a weekend* ***I can edge for hours***." | 30M | PPU forum |
| - "*I usually only orgasm once a day and I know I'm going to regret it when I finish, so* ***I try to masturbate and look at porn for as long as I possibly can***/" | 25M | PPU forum |
| - "*At times I've watched porn from an afternoon well into the late night.* ***I typically have these binges 1-3 times a fortnight***." | 23M | Social media |
| - "*I have had binge experiences where I would either* ***watch porn for maybe 2 or 3 hours, or masturbate many times a day****. When binging porn in long sessions (assuming I have held ejaculation), the orgasm is very strong. However in both cases, sensitivity decreases in later sessions. Nevertheless, the number of orgasms is equal to the number of sessions, although the intensity of it is decreased*." | 19M | Social media |
| - "***12-24h, sometimes going on and off for days****. The first time I didn't sleep due to a porn binge was after ~ 10 years of use.* ***Nowadays it happens almost every week*** *that I lose 1-2 nights of sleep due to binges. It is a big problem, even just due to the lack of sleep*" | 29M | PPU forum |
| - “*I believe I once had a day where* ***I orgasmed 12 times in a day, sensitivity diminished each time, but porn kept me going****, and I would do this every other weekend or so... Not 12 in a day, but at least 5.*” | 27M | PPU forum |
| - "*Novelty is important. I get bored quickly and want to move on. I frequently move between different content to find the "best" one. But I'm never satisfied.* ***The search can be hours long***." | 26M | PPU forum |
| - "*Often on weekends or after work,* ***I can binge for several hours. I will 'edge' to near the point of climax, and stop for a moment, and continue again****. Eventually after a period of time,* ***sometimes several hours, I will finish***." | 27M | Social media |
| - "*Usually* ***jerking multiple times a day, sometimes as many as 5****. It becomes slightly more difficult each time to finish*." | 22M | Social media |
| - "***I'll watch it all day if I'm home alone****."* | 35M | Social media |
| - "*A couple of months ago, less frequently, but* ***now, every day 3-4 times a day, for 1-2 orgasms each****.*" | 19M | PPU forum |
| - "***Mostly edging. Orgasm maybe 4 or 5 times a day.***" | 22M | PPU forum |
| - "*I would* ***masturbate multiple times a day maybe like 3-8 times*** *and by the end of the day my sensitivity will be gone and I will be feeling extra depressed*" | 20M | PPU forum |
| - "*It's been many years, but* ***if I knew the house would be empty for a while I would binge porn****, explore different types of porn while masturbating*" | 54M | PPU forum |
| - Binge aftermath: "*I usually feel mentally drained and fatigued, also feel lots of shame & embarrassed that I've returned to porn"* | 23M | Social media |
| - “*I used to* ***binge for 4+ hours more than twice a week****. Now maybe once a month. Only one orgasm at a time, I never felt the need to keep going after one. It's like a blur of positive feelings, and the time passes really quickly*." | 27M | Social media |
| - "***Hours of trying to get off****. Sensitivity and response to stimulation decreases. You’re left feeling frustrated*." | 21M | Social media |
| - "***Binges typically include 3 or 4 wanks a day across a 2-3 day period*** *before I am completely depleted and have nothing left to give*." | 18M | Social media |
| - "***Can last for hours where I find myself moving between different material, orgasming multiple*** *times. I'm not sure what triggers this however it normally ends when I am tired or too sore to continue*." | 32M | Social media |
| - “*Usually my binges will run closer together and I might* ***orgasm a few times over 1-2 hours***." | 23F | Social media |
| - “***I will watch porn for 2 hours straight sometimes****, just mindlessly scrolling through whatever platform I'm using it*." | 22F | Social media |
| - "*Viewing for around* ***20 hours, 4 orgasm[s], slightly lowered sensitivity****.*" | 25M | Social media |
| - "*I have masturbated with porn* ***5-6 times a day, multiple consecutive days before****.* ***I have done 3-4 hour long sessions till 2am at night to porn****. The longer sessions are actually far more sexually pleasing than normal, with* ***edging and the final orgasm being some of, if not the best, orgasms I've had in my life***." | 22M | Social media |
| - "***The more binges I have, the stranger and the more kinks I get***." | 23M | Social media |

Note: Age_NS_ = age not specified, NC = non-cisgender
